# Influence of Hydroxyapatite Surface Functionalization on Thermal and Biological Properties of Poly(l-Lactide)- and Poly(l-Lactide-co-Glycolide)-Based Composites

**DOI:** 10.3390/ijms21186711

**Published:** 2020-09-13

**Authors:** Małgorzata Gazińska, Anna Krokos, Magdalena Kobielarz, Marcin Włodarczyk, Paulina Skibińska, Bogusz Stępak, Arkadiusz Antończak, Milena Morawiak, Przemysław Płociński, Karolina Rudnicka

**Affiliations:** 1 Department of Engineering and Technology of Polymers, Faculty of Chemistry, Wrocław University of Science and Technology, Wyb. Wyspiańskiego 27, 50-370 Wrocław, Poland; milena.morawiak@pwr.edu.pl; 2 Department of Mechanics, Materials and Biomedical Engineering, Faculty of Mechanical Engineering, Wroclaw University of Science and Technology, Wyb. Wyspiańskiego 27, 50-370 Wrocław, Poland; magdalena.kobielarz@pwr.edu.pl; 3 Department of Immunology and Infectious Biology, Faculty of Biology and Environmental Protection, University of Łódz, Banacha 12/16, 90-237 Łódź, Poland; marcin.wlodarczyk@biol.uni.lodz.pl (M.W.); paulina.skibinska@biol.uni.lodz.pl (P.S.); przemyslaw.plocinski@biol.uni.lodz.pl (P.P.); karolina.rudnicka@biol.uni.lodz.pl (K.R.); 4 Laser and Fibre Electronics Group, Faculty of Electronics, Wrocław University of Science and Technology, Wyb. Wyspiańskiego 27, 50-370 Wrocław, Poland; bogusz.stepak@pwr.edu.pl (B.S.); arkadiusz.antonczak@pwr.edu.pl (A.A.)

**Keywords:** biocomposites, hydroxyapatite surface biofunctionalization, l-lysine, biosafety, monocytes

## Abstract

Novel biocomposites of poly(L-lactide) (PLLA) and poly(l-lactide-co-glycolide) (PLLGA) with 10 wt.% of surface-modified hydroxyapatite particles, designed for applications in bone tissue engineering, are presented in this paper. The surface of hydroxyapatite (HAP) was modified with polyethylene glycol by using l-lysine as a linker molecule. The modification strategy fulfilled two important goals: improvement of the adhesion between the HAP surface and PLLA and PLLGA matrices, and enhancement of the osteological bioactivity of the composites. The surface modifications of HAP were confirmed by attenuated total reflectance-Fourier transform infrared spectroscopy (ATR-FTIR), TGA, and elemental composition analysis. The influence of hydroxyapatite surface functionalization on the thermal and in vitro biological properties of PLLA- and PLLGA-based composites was investigated. Due to HAP modification with polyethylene glycol, the glass transition temperature of PLLA was reduced by about 24.5 °C, and melt and cold crystallization abilities were significantly improved. These achievements were scored based on respective shifting of onset of melt and cold crystallization temperatures and 1.6 times higher melt crystallization enthalpy compared with neat PLLA. The results showed that the surface-modified HAP particles were multifunctional and can act as nucleating agents, plasticizers, and bioactive moieties. Moreover, due to the presented surface modification of HAP, the crystallinity degree of PLLA and PLLGA and the polymorphic form of PLLA, the most important factors affecting mechanical properties and degradation behaviors, can be controlled.

## 1. Introduction

Tissue engineering belongs to an interdisciplinary research field that combines biological and engineering knowledge. The goal of tissue engineering is the repair or replacement of failing organs or tissues with the use of biological substitutes that can restore, support or improve tissue functions [1,2]. Hydroxyapatite (HAP) can be used in hard tissue regeneration due to its chemical similarity to the mineral phase of the bone and tooth [3]. Synthetic hydroxyapatite has been widely investigated as the major, inorganic component of scaffolds for applications in bone tissue engineering [1].

Natural bone is a rather sophisticated structure composed of mineral and organic phases. The hydroxyapatite phase is embedded in an extracellular matrix primarily composed of collagen and is surrounded by living tissues [4]. Whereas collagen is the main structural protein, bones are infused with other proteins, like growth factors, chemokines, numerous extracellular matrix proteins and regulatory proteins to orchestrate bone homeostasis. The bone-forming osteoblasts are counteracted by bone-resorbing osteoclasts, which together maintain the state of constant remodeling that is typical for this type of tissue [5]. To promote healing, tissue-engineered bone regeneration matrices have to form a scaffolding niche that provides a similar balance between strength and toughness as the natural bone. This niche must also be readily populated by appropriate cell types that are non-toxic and reabsorbable. Functionalization of the biocomposites with proteins or amino acids helps to mimic the natural environment of the human body and increases the biocompatibility of the synthetic materials.

The growth and remodeling of the bone tissue are connected to the function of the immune system. The constant resorption of the bone is driven by osteoclasts, which differentiate from hematopoietic progenitor cells of the monocyte/macrophage lineage and are considered as highly specialized immune cells. Bone forms a unique niche where the immune cell progenitors and memory cells reside and proliferate. Notably, osteoclast cell differentiation is driven via the same Receptor Activator for Nuclear Factor-κB Ligand (RANKL) pathway that is critical for the function of various immune cells. Most importantly, numerous inflammatory disorders may tremendously affect bone formation and the healing of bone fractures, signifying the importance of the interplay between bone tissue and the immune system [6]. Hence, any tissue-engineered materials for bone regeneration must not be immunogenic and must not promote inflammation at the site of grafting.

Biocomposites based on HAP particles and bioresorbable polymers, such as poly(l-lactide) (PLLA) and poly(l-lactide-co-glycolide) (PLLGA), improve the biological response of bone-forming cells and may significantly enhance the biomineralization process. The main advantages of using such biocomposites are their biocompatibility, biodegradability, and good osteoconductive and osteoinductive properties [2,7].

In this study, we aimed to investigate the influence of hydroxyapatite particles’ surface modification on the thermal and biological properties of composites based on PLLA and PLLGA. The innovation of the study was the hydroxyapatite surface modification strategy. The modification was designed to fulfill two important goals: firstly, to improve the compatibility of the composite components by increasing the adhesion between the HAP surface and PLLA and PLLGA matrices and secondly, to increase the osteological bioactivity of composites that are dedicated to regenerative medicine for bone regeneration. To achieve miscibility at the molecular level, polyethylene glycol (PEG) of varying molecular weights was used for modification of the surface of hydroxyapatite particles. PEG is miscible with PLLA, with the miscibility limit depending on its molecular weight. Additionally, PEG having an appropriate molecular weight plays the role of PLLA plasticizer [8,9]. According to the presented modification pathway, PEG is grafted on the surface of HAP with the assistance of l-lysine. In grafting procedures, toxic chemical linkers such as diisocyanates or (3-aminopropyl) triethoxysilane are typically used. In place of such commonly used reagents, l-lysine was proposed to assist PEG grafting and to improve osteological bioactivity of the composites. l-lysine is known to promote the adhesion of osteoblasts to the surface of the material, which may positively correlate with cell proliferation [10,11], and was found to improve the osteogenic potential of the bone marrow stromal cells [12]. l-lysine can be used as a food supplement and its intake benefits in increased bone mineral density and lowered prevalence of osteoporosis [13]. As per recent literature reports, a surface with controlled amino acid density and organization could be used as a tool for regulating HAP precipitation. Understanding the effect of single amino acids could lead to rationally designed oligopeptides to tune the mineralization process [14]. In these terms, we intentionally employed l-lysine as a nanoscale linker molecule in the grafting procedure.

The HAP particles consist of a Ca^2+^-rich region on their surfaces. The Ca^2+^ site of HAP binds to the negatively charged anionic groups such as carboxyl COO^−^. It thus provides the ability of incorporation of biological molecules such as amino acids to the surface of HAP [3]. Qualitative and quantitative analyses of l-lysine molecules incorporated on the HAP surface and the interaction mechanism were described by Kollath et al. [15].

To increase the compatibility between the filler surface and polymer matrix, grafting of molecules miscible with polymers onto the filler particles is an effective solution. Thus, in the proposed surface modification pathway, a well-known PLLA plasticizer was used. Among other known PLLA plasticizers, such as epoxidized palm oils, polypropylene glycol, citrate esters, oligomeric malonate esteramides, polyglycerol esters, poly(1,3-butylene adipate), glucose monoesters, and oligomeric lactic acid [9], polyethylene glycol was selected based on its biosafety for medical applications. Polyethylene glycol is non-immunogenic, non-antigenic, and non-toxic, and does not harm active proteins or cells although it interacts with cell membranes. These properties enable a variety of important biotechnical and biomedical applications [16].

The range of applicability of PEGs as PLLA plasticizers is given in terms of PEG molecular weight and its concentration in PLLA/PEG blends. Baiardo et al. [17] plasticized PLLA using PEGs with different molecular weights (M_w_ = 400–10,000 g/mol) and discovered that lower molecular weight PEG had better plasticizing efficiency in the PLLA matrix and more significantly reduced the glass transition temperature (T_g_) (from 60 °C in pure PLLA to 19 °C in a PLLA/PEG blend with 20 wt.% PEG400). They also found the solubility limits, which moved toward lower PEG concentration with increasing molecular weight (30 wt.% for PEG400 and 15 wt.% for PEG10000). Li et al. [18] also plasticized PLLA using PEGs with five different molecular weights in the range of 200–20,000 g/mol. The scanning electron microscope results confirmed the better compatibility and therefore more efficient plasticizing effect of lower molecular weight PEGs (such as from 200 to 6000 g/mol) on PLLA. T_g_ decreased from 59.2 °C in pure PLLA to 45.7 °C in PLLA/PEG blend with 5 wt.% PEG10000. For blends containing higher concentrations of PEG1000, glass transition temperature was indistinguishable on DSC curves. However, the most efficient plasticizing effect, confirmed by both the V-notched and Charpy impact strengths, was obtained for PLLA/PEG10000 blend with 20 wt.% plasticizer. Yu et al. [19] described similar results for the plasticizing effect of PEGs with different molecular weights from the range of 300–10,000 g/mol in PLLA/starch blends. The PEG with the lowest molecular weight in the research (300 g/mol) reduced T_g_ with the highest efficiency, from 62 °C in pure PLLA to 29 °C in PLLA/starch/PEG blend with 13 wt.% PEG300. The crystallinity degree of this blend increased to 30.9% (from 6.3% in pure PLLA). The high crystallinity of the PLLA matrix and lowered T_g_ can be attributed to the effective plasticization of PEGs.

Here, we introduced hydroxyapatite modified with l-lysine and PEG into a PLLA homopolymer and PLLGA copolymer. To the best of our knowledge, composites of PLLA and PLLGA containing HAP grafted with linear polyethylene glycol with the assistance of l-lysine have not yet been described in the literature. Concerning the potential of practical applications of investigated composites in regenerative tissue engineering, the HAP was modified using chemical compounds approved for biological applications. The main goal of this research was to design novel biocomposites based on PLLA and PLLGA, and multifunctional hydroxyapatite particles dedicated for bone regenerative applications. The impact of hydroxyapatite surface modification on thermal properties, such as crystallinity degree and melt and cold crystallization behaviors of PLLA and PLLGA, was also determined.

As the proposed modification pathway is unknown, standard direct contact cytotoxicity studies were performed toward L929 fibroblasts. Apart from recommended in vitro studies, the viability of osteoblasts (hFOB 1.19) cultured in the milieu of the composites was also evaluated. To exclude potential pathological immunostimulatory potential of composites, the THP1-Blue^TM^ biosensing system was used to perform a screening assay for the nuclear factor kappa-light-chain-enhancer of activated B cells (NF-ĸB) transcription factor induction.

## 2. Results and Discussion

### 2.1. Characterization of Hydroxyapatite Surface Modification

**Figure 1:**
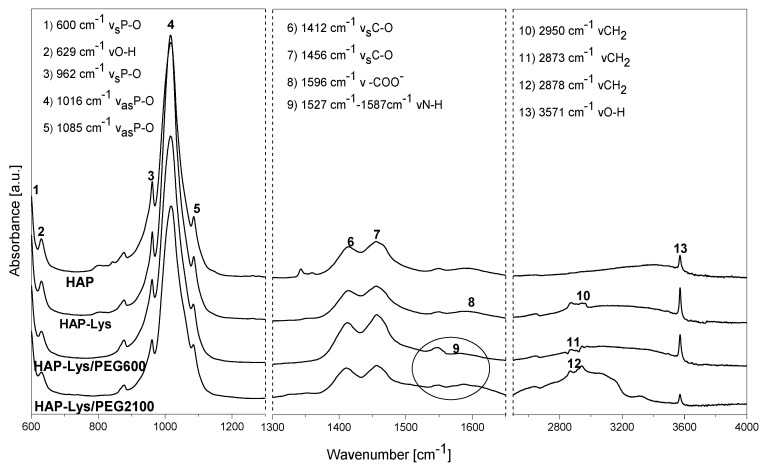
**The attenuated total reflectance-Fourier transform infrared** (FTIR-ATR) spectra of unmodified hydroxyapatite (HAP), HAP modified with l-lysine, and HAP after polyethylene glycol (PEG) grafting.

FTIR analysis. Figure 1 presents the attenuated total reflectance-Fourier transform infrared (ATR-FTIR) pectra of unmodified HAP particles, HAP with surface modified with l-lysine and HAP after PEG grafting. In the HAP spectrum, the intense bands at 1016 and 1085 cm^−1^ corresponded to P–O asymmetrical vibration modes (ν_3_). The doublet at 600 cm^−1^ was assigned to the P–O stretching mode (ν_4_). The weak band at 962 cm^−1^ corresponded to the stretching vibration mode (ν_1_) of the P–O bond in PO^4−^ groups. The absorption bands observed at 3571 and 629 cm^−1^ corresponded to the stretching and deformation vibrations of O–H bonds in the hydroxyl groups of the crystalline phase of hydroxyapatite, respectively. The absorption band at approximately 3400 cm^−1^ and the wide and weak absorption band at approximately 1600 cm^−1^ were assigned to water adsorbed on the surface of the HAP. In the spectrum of HAP-Lys, the presence of bands at 2950 cm^−1^ from the alkane chain of l-lysine indicated the presence of an amino acid and the band at 1596 cm^−1^ confirmed the formation of carboxylate on the surface of HAP [15]. Moreover, the bands at 1575, 2873, and 2950 cm^−1^ (alkane chain of lysine) were shifted in the case of HAP-Lys compared to the position of pure l-lysine bands, indicating the existence of a weak interaction of l-lysine with HAP. The FTIR-ATR spectrum of pure l-lysine combined with FTIR-ATR spectra of HAP and HAP-Lys is presented in Figure S1 in the Supplementary Materials. For HAP particles after PEG grafting, the absorption bands at 2878 cm^−1^ (HAP-Lys/PEG2100) and 2873 cm^−1^ (HAP-Lys/PEG600) corresponded to the C–H stretching vibration mode of the alkanes group and indicated the presence of l-lysine and polyethylene glycol on the surface of HAP. The band at around 1262 cm^−1^ corresponded to C–O–C vibration from PEG. The formation of an amide bond in the reaction of the carboxyl end group of PEG and the first order amine group of l-lysine was confirmed by the characteristic second amide band in the range of 1587–1527 cm^−1^ from the deformation vibration of the N–H bond [15,20].

**Figure 2:**
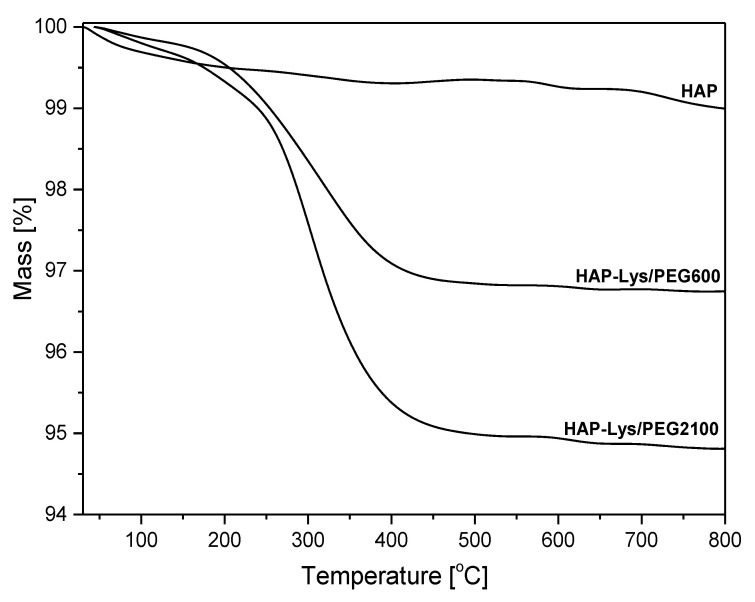
TGA curves of hydroxyapatite, hydroxyapatite modified with l-lysine and PEG600 (HAP-Lys/PEG600), and hydroxyapatite modified with l-lysine and PEG2100 (HAP-Lys/PEG2100).

TGA analysis. The total amounts of grafted l-lysine and PEGs onto the HAP surface were determined by thermogravimetric analysis. According to the TGA curves presented in Figure 2, the weight losses at 800 °C of unmodified HAP and HAP modified with l-lysine/PEG600 and l-lysine/PEG2100 were 0.927%, 3.178%, and 5.007%, respectively. PEG600, PEG2100, and l-lysine degraded completely up to 800 °C (Figure S2 in the Supplementary Materials). The surface-grafted amount on the HAP surface was calculated from the residue at 800 °C considering the 0.927% of mass loss of HAP. The total grafting amount on HAP modified with l-lysine/PEG600 was found to be 2.251%, and 4.080% for HAP modified with l-lysine/PEG2100. This result further proved that l-lysine and PEG600/PEG2100 were successfully grafted onto the HAP surface. It also indicated the higher amount of grafted PEG2100 than PEG600. The estimated higher content of PEG2100 agreed with the amount assumed in the grafting procedure described in Section 3 and stemmed from the higher PEG2100 amount required for grafting than PEG600 with the maintained equal molar ratio.

### 2.2. Morphology Characterization of Hydroxyapatite Particles and Dispersion Effect in Composite Films

**Figure 3:**
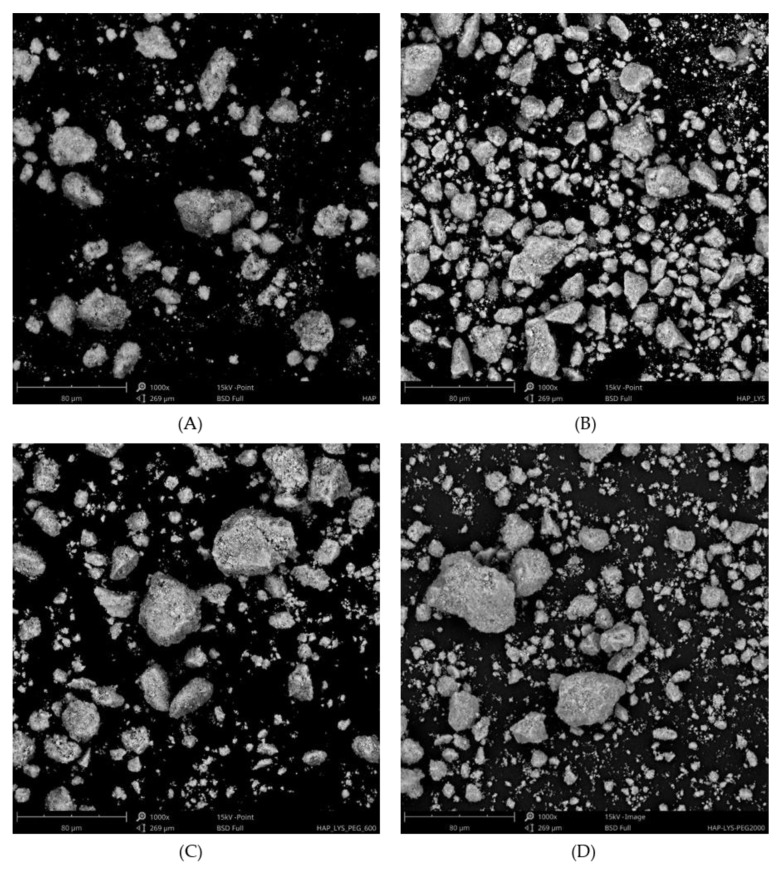
SEM images of (**A**) HAP, (**B**) HAP-Lys, (**C**) HAP-Lys/PEG600, and (**D**) HAP-Lys/PEG2100.

The SEM images in Figure 3 show that hydroxyapatite consisted of particles composed of needle-shaped crystallites. Modifications of HAP by polyethylene glycol and l-lysine did not influence the surface morphology. The interface between inorganic hydroxyapatite and organic phases, for both PEG and l-lysine, was indistinguishable mainly due to the high affinity of inorganic crystals to the polymer matrix of PEG [21]. The quantitative analysis, performed to calculate the average size of particles, revealed a notable morphological difference between pure and functionalized HAPs (Table 1). For HAP powder, more aggregation was noted in comparison to functionalized hydroxyapatite. Organic compounds can probably inhibit the agglomeration of inorganic nanomaterials. However, for HAP-Lys (Figure 3B) and HAP-Lys/PEG2100 (Figure 3D), considerable spread of powder particle size was observed, while for HAP-Lys/PEG600 (Figure 3C), particle aggregation was the lowest, resulting in the lowest average particle size. A lower aggregation level of HAP-PEG particles in composites was reported by Akhilesh et al. [22]. Well-dispersed particles were also identified for hydroxyapatite functionalized by l-lysine. More dispersed and less aggregated particles of functionalized HAP are more appropriate for use in biomedical applications.

The atomic percentage of nitrogen (at%) indicated a high amount of linker molecules, above 6% and 8% for HAP-Lys and HAP-Lys/PEG (600 and 2100), respectively, present on the surface of the modified hydroxyapatite. These results agreed with that of the TGA. However, the distribution of nitrogen on the HAP surfaces was heterogeneous because there were areas with undetected nitrogen. Peaks of Ca and P in the EDS spectra of hydroxyapatite samples with and without functionalization were highly pronounced and exhibited a framework similar to that of standard hydroxyapatite [23]. Table 1 lists the calcium and phosphorus contents in the studied powders. The molar ratio of calcium to phosphorus in the standard stoichiometric hydroxyapatite is 1.67 [24]. The Ca:P molar ratio obtained from quantitative analysis for HAP was congruent and slightly higher for all functionalized HAP samples; however, all fell within the acceptable range of 1.4–2.4 for the atomic Ca:P ratio of stable hydroxyapatite [25]. A quantitative analysis of carbon should be considered cautiously due to the risk of contamination of adventitious carbon originating from carbon pads; hence, it could not be used as a basis for assessing the attachment of organic compounds to the HAP.

**Figure 4:**
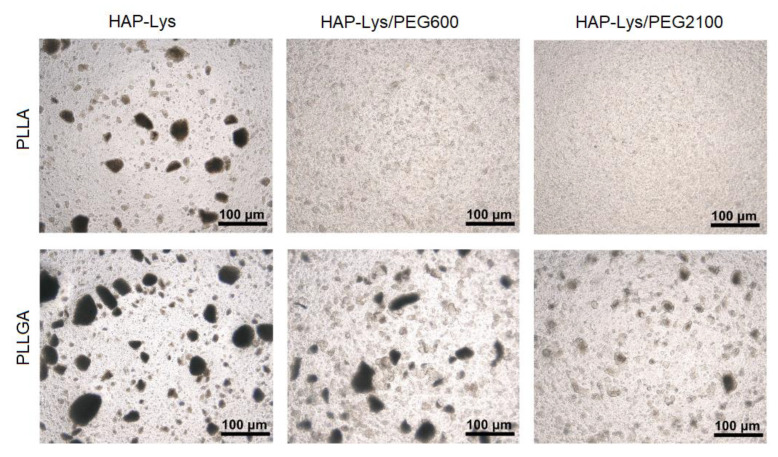
Optical images of composite films.

The composite samples were illuminated form the back side under an optical microscope. The same illumination conditions were applied for all the samples. In Figure 4, hydroxyapatite agglomerates can be seen with the size differing depending on the type of HAP surface modification and polymer used as the matrix. The brightness of agglomerates revealed their thickness. Surface modification of HAP-Lys particles with PEG improved the homogeneity of particle distribution in a polymer matrix and resulted in significant reduction of the dispersed agglomerate size. The improvement of homogeneity was more prominent in PLLA than in PLLGA. PEG2100-modified HAP-Lys produced a greater size reduction in hydroxyapatite agglomerates than PEG600 with the PLLA polymer, whereas for PLLGA, the influence of PEG molecular weight was not as perceptible.

### 2.3. Water Contact Angle of PLLA, PLLGA, and Composite Film

**Figure 5:**
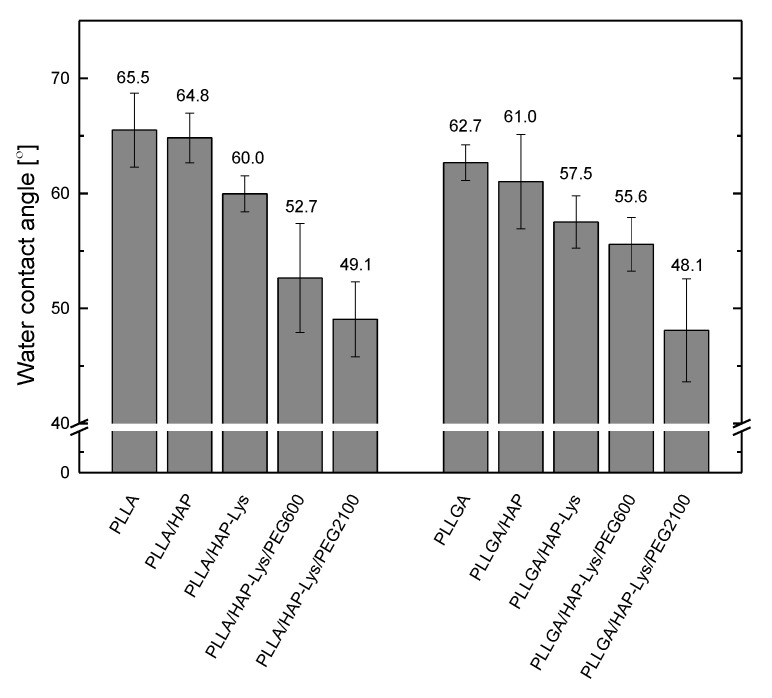
Water contact angle of poly(l-lactide) (PLLA), poly(l-lactide-co-glycolide) (PLLGA), and composite films.

Water contact angle measurements are presented in Figure 5. The results revealed decreasing water contact angle of PLLA- and PLLGA-based composite films in the presence of filler particles in the following order: HAP, HAP-Lys, HAP-Lys/PEG600, and HAP-Lys/PEG2100. For neat PLLA, the water contact angle was 65.5 ± 3.2°C and decreased to 49.1 ± 3.3°C for PLLA/HAP-Lys/PEG2100. This represented an increase in hydrophilicity due to the hydrophilic mineral filler (water contact angle for HAP is ~10° [26]) and polyethylene glycol in composite films. The results indicated a significant improvement in wettability of PLLA- and PLLGA-based composites with PEG-modified HAP, which is important in terms of future application of the composites for bone regeneration.

### 2.4. DSC Results of PLLA and PLLA-Based Composites

**Figure 6:**
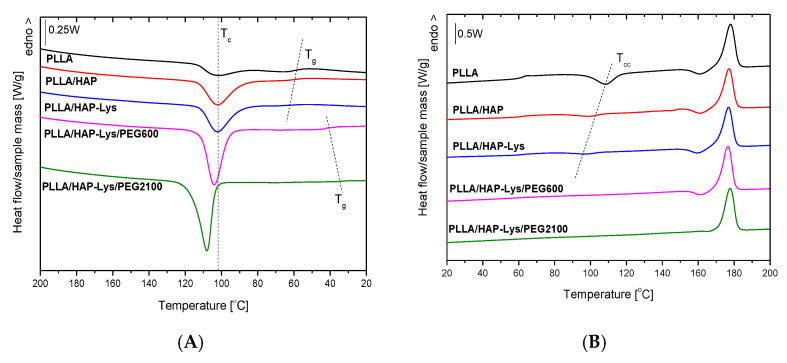

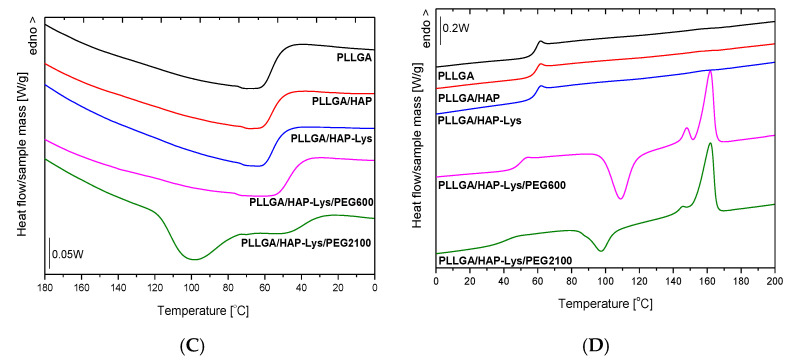
The cooling and second heating DSC curves of (**A**,**B**) PLLA and PLLA-based composites and (**C**,**D**) PLLGA and PLLGA-based composites.

To investigate the effect of hydroxyapatite surface modification with l-lysine and polyethylene glycol on the thermal properties of PLLA- and PLLGA-based composites, DSC experiments were performed. The composite samples were first heated to 200 °C and maintained at 200 °C for 3 min under nitrogen to erase previous thermal history. The cooling DSC curves of the PLLA sample series are shown in Figure 6A, and the estimated thermal parameters are presented in Table 2. During cooling from the melt, PLLA underwent crystallization with an onset at 114.1 °C. The enthalpy of melt crystallization (ΔH_c_) occurring with a cooling rate of 5 °C/min had the lowest values for neat PLLA (11.8 J/g). In the presence of unmodified and l-lysine-modified hydroxyapatite particles, the enthalpy of melt crystallization increased; however, crystallization started 1.3 °C and 2.1 °C lower, respectively. In composites with HAP modified with PEG600 and PEG2100, the increase in ΔH_c_ was much more prominent, 1.35 and 1.6 times higher than neat PLLA, respectively. For HAP modified with PEG2100, onset of melt crystallization (T_c_^onset^) moved about 1.5 °C higher, with a narrower temperature range of melt crystallization. These results indicated the nucleating activity of HAP modified with PEGs, with a higher efficiency of melt crystallization nucleation exhibited by HAP modified with PEG2100.

The glass transition temperature from the cooling scan of neat PLLA, determined as the inflection point of the heat capacity drop, was 59.7 °C. PLLA in composites with HAP modified with PEG600 exhibited a T_g_ about 16 °C lower. A more efficient reduction of T_g_ (about 24.5 °C) was attained for PLLA with HAP modified with PEG2100.

The glass transition temperature decrease and increase in melt crystallization temperature indicate the plasticizing action of PEGs. To assess the plasticization effectiveness of PEG2100 and PEG600, several factors were considered. The effectiveness of plasticization depends on the molecular weight of PEG, showing an enhanced effect with lower molecular weight PEG [8]. In our case, the higher plasticization efficiency of PEG2100 compared with PEG600, indicated by lower T_g_ and higher peak temperature of melt crystallization (T_c_) and T_c_^onset^, could steam from its higher weight content due to the grafting procedure applied, as confirmed by TGA.

The glass transition temperature of semicrystalline PLLA also depends on morphology. A lower T_g_ was exhibited by PLLA with a lower degree of crystallinity due to having a lower content of rigid amorphous fraction. Larger amounts of rigid amorphous fraction result in a higher T_g_ [27].

It should also be emphasized that the two applied carboxyl-terminated PEGs, PEG2100 and PEG600, have different chemical structures. PEG2100 is a succinylated monomethoxypolyethylene glycol, whereas PEG600 is carboxylic acid functionalized methoxy undecaethylene glycol. The effect of different end groups in PEGs on the plasticization of PLLA is described in the literature. The influence of PEG terminated with two hydroxyl end groups in comparison to two methyl end groups in PEG with a molecular weight of 2100 g/mol was reported for a PEG concentration of 70 wt.% [28]. The equilibrium melting temperature decrease was greater for PEG terminated with methyl groups than hydroxyl groups. PEG terminated with two hydroxyl groups and PEG with one hydroxyl group substituted by a methyl group were examined by Kulinski et al. [8]. No clear influence of the PEG plasticizer end group on the thermal properties of plasticized PLLA was found for PEG content not exceeding 10 wt.%; the PLLA properties were influenced by plasticizer content.

The second heating DSC curves are presented in Figure 6B and the estimated thermal parameters are presented in Table 3. On second heating at temperatures above T_g_, the exothermic peak of cold crystallization was visible for PLLA, PLLA with unmodified HAP, and l-lysine-modified HAP particles. The enthalpy of cold crystallization (ΔH_cc_) was lower for PLLA with HAP particles than for neat PLLA because a crystalline phase partially formed on cooling in the presence of HAP particles. Lowering the onset of cold crystallization (T_cc_^onset^) and peak temperature of cold crystallization (T_cc_) indicated the facilitated crystallization of PLLA in the presence of HAP particles.

The lack of cold crystallization of PLLA in composite with HAP modified with PEG600 as well as PEG2100 indicated that crystallization of PLLA was complete on cooling due to the high nucleating efficiency of modified HAP particles. The difference between HAP modified with PEG600 and PEG2100 concerned the existence of α’-α phase transition, visible before the main melting peak. Prior to the melting peak, a small exothermic effect was observed for PLLA, PLLA/HAP, PLLA/HAP-Lys, and PLLA/HAP-Lys/PEG600. This peak indicated the phase transition of crystalline α’ phase to the thermodynamically favored α form [29]. The absence of an exothermic effect of the α’-α phase transition for PLLA with HAP modified with PEG2100 indicated that PLLA crystallized on cooling in α form. HAP modified with PEG2100 nucleated the α phase crystallization compared with PEG600.

The T_g_ of PLLA from the second heating scan was 62.5 °C. In PLLA/HAP-Lys/PEG600 and PLLA/HAP-Lys/PEG2100 composites, T_g_ was lowered to 57 °C and 46 °C, respectively. This finding confirmed the results obtained from the cooling scan. The melting temperatures (T_m_) of composites containing surface-modified hydroxyapatite were also lower than the T_m_ of PLLA (177.9 °C) by about 7 °C and 5.9 °C for PLLA/HAP-Lys/PEG600 and PLLA/HAP-Lys/PEG2100, respectively.

The decreased glass transition temperatures with increasing crystallinities in composites containing hydroxyapatite modified with l-lysine and PEG600/PEG2100 highlighted the significant plasticizing effect reached at low PEG content due to the surface modifications of HAP. As summarized by Saeidlou et al. [30], the addition of PEG reduces the T_g_ by approximately 2 °C per 1 wt.% plasticizer. The T_g_ of PLLA was reduced by 24.5 °C for HAP modified with PEG2100 and by 16.0 °C in the composite with HAP-Lys/PEG600, with a PEG content not exciding 5 wt.%.

### 2.5. DSC Results of PLLGA and PLLGA-Based Composites

Cooling curves of PLLGA sample series are presented in Figure 6C. Neither HAP modified with PEG600 nor PLLGA and PLLGA with unmodified and l-lysine-modified HAP particles were able to crystallize from melt during cooling with the scanning rate employed (5 °C/min). The exothermic effect of melt crystallization was only visible for PLLGA/HAP-Lys/PEG2100, indicating the nucleation activity of HAP participles modified with PEG2100 on melt crystallization of PLLGA. HAP modified with PEGs caused a decrease of glass transition. PLLGA with HAP modified with PEG2100 exhibited a T_g_ about 17.7 °C lower (at 38.7 °C) than neat PLLGA. Plasticizing activity was also demonstrated by HAP modified with PEG600, but with lower efficiency than PEG2100; a decrease of T_g_ of about 9.4 °C was reached. The lowering of glass transition temperature by HAP modified with PEG600 and PEG2100 was confirmed by the second heating DSC curves (Figure 6D). PLLGA and PLLGA with unmodified and l-lysine-modified HAP particles did not crystallize on heating. The cold crystallization effect was visible for PLLGA with HAP modified with PEG600 and PEG2100. HAP modified with PEG600 did not exhibit nucleation activity on melt crystallization, but effectively nucleated the cold crystallization of PLLGA. A higher efficiency of cold crystallization nucleation was exhibited by HAP with PEG2100, as indicated by lower values of onset T_cc_^onset^ as well as peak temperature T_cc_ of cold crystallization.

### 2.6. In Vitro Cytocompatibility of PLLGA and PLLGA-Based Composites

**Figure 7:**
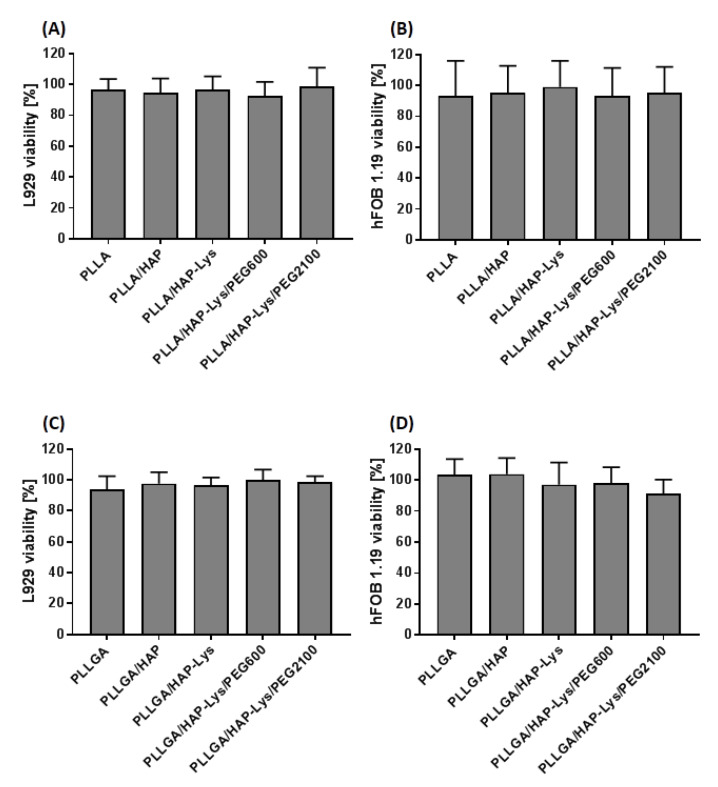
Viability of (**A**,**C**) murine fibroblasts L929 and (**B**,**D**) human osteoblasts hFOB 1.19, incubated for 24 h with (**A**,**B**) PLLA- or (**C**,**D**) PLLGA-based composites, evaluated using the 3-(4,5-dimethylthiazol-2-yl)-2,5-diphenyltetrazolium bromide (MTT) reduction assay according to ISO-10993-5:2009. The cells incubated without composites served as a positive control of viability (100%). Data are presented as mean ± SD of three separate experiments (six replicates for each assay ).

PLLA and PLGA have been considered as two of the most potent candidates for biomedical applications [31,32]. Due to their cytocompatibility, biodegradable properties, non-toxicity, and mechanical features, they are extensively used to fabricate tissue engineering scaffolds [33]. The biocomposites for medical applications destined for implantation or contact with tissue for long periods had to meet particular in vitro cytocompatibility criteria, even in the early stages of composite optimization (ISO 10993-5:2009). In this study, apart from standard L929 mouse fibroblasts, which are recommended by ISO standards, we used hFOB 1.19 human osteoblasts, that mimic the environment in which composites designed for bone regeneration stay in contact with. We found that the viability of target cells exposed to PLLA and PLLGA composites and their modifications remained at the same level as untreated controls. The results of the 3-(4,5-dimethylthiazol-2-yl)-2,5-diphenyltetrazolium bromide (MMT) reduction assay, which determines the metabolic function of cells by measuring the activity of mitochondrial dehydrogenase, showed that none of the PLLA- or PLLGA-based composites exhibited cytotoxicity after direct contact with these materials. As shown in Figure 7A, the viabilities of murine fibroblasts exposed to PLLA, PLLA/HAP, PLLA/HAP-Lys, and PLLA/HAP-Lys/PE600 were found to be 96.6% ± 6.8%, 94.6% ± 9.1%, 96.6% ± 8.4%, 92.4% ± 9.2%, and 98.6% ± 12.2%, respectively. The modifications of composites based on PLLGA (PLLGA, PLLGA/HAP, PLLGA/HAP-Lys, PLLGA/HAP-Lys/PEG600, and PLLGA/HAP-Lys/PEG2100) did not influence the viability of L929 cells (93.9% ± 8.4%, 97.6% ± 7.4%, 96.5% ± 5.1%, 99.9% ± 6.7%, and 98.3% ± 4.0%, respectively; Figure 7C). A similar effect was observed when hFOB 1.19 osteoblasts were exposed to PLLA-based composites (93.4% ± 22.5%, 95.2% ± 17.5%, 98.9% ± 17.0%, 93.2% ± 18.0% and 95.1% ± 16.9% for PLLA, PLLA/HAP, PLLA/HAP-Lys, PLLA/HAP-Lys/PEG600, and PLLA/HAP-Lys/PEG2100, respectively; Figure 7B) as well as PLLGA-based composites (103.6% ± 9.9%, 103.7% ± 10.5%, 97.0% ± 14.2%, 98.1% ± 10.2%, and 98.1% ± 9.3% for PLLGA, PLLGA/HAP, PLLGA/HAP-Lys, PLLGA/HAP-Lys/PEG600, and PLLGA/HAP-Lys/PEG2100, respectively; Figure 7D). All composites met the ISO criterion of maintaining the viability of at least 70% of cells exposed to the biomaterial for 24 h (ISO 10993-5:2009). A statistical analysis showed that modifications of PLLA-based and PLLGA-based composites did not affect the viability of target cells when compared with the physiological viability of control cell cultures. These results were in line with findings observed by other authors. Surfaces functionalized by arginine-glycine-aspartic acid and lysine-arginine-serine-arginine were found to support attachment, but did not enhance osteoblast differentiation or proliferation rate [34]. However, functionalization of surfaces with specific peptides, including l-lysine, may favor the attachment of osteoprogenitor cells and inhibit osteoblastic differentiation. The supporting impact of l-lysine on the attachment and viability of cells in the milieu of medical biomaterials was also proven. Recently, Castillo-Cruz et al. developed a Lys-based hydrogel that enhanced cell adhesion due to lysine residues. It was observed that the attachment of cells to lysine-modified hydrogel was five times more effective, compared with a poly-l-lysine coating. The interaction between the biomaterial and the cells might occur through electrostatic interaction between the positive charge from –NH^3+^ and negatively charged substances on the cell surface such as glycosaminoglycans [35,36]. The synergistic role of l-lysine and hydroxyapatite in osteoblast healing processes was also reported [11]. The results presented here as well as findings of other groups suggests that PLLA and PLGA with l-lysine modified hydroxyapatite support the cytocompatibility of composites, and that the biological activity remains when facilitating the adhesion process, which drives regeneration rather than cell proliferation or metabolic activity.

### 2.7. Immunocompatibility of THP1-Blue™ NF-κB Human Monocytes with PLLA and PLLGA Composites

Monocytes are among the most sensitive cells that upon activation rapidly induce the inflammatory cascade. The controlled inflammation leads to revascularization and regeneration of tissue at injury sites. However, strong activation of monocytes by the components of the biomaterial or contaminants such as endotoxins induce acute inflammation that results in pus formation, tissue degradation, and disintegration of cell barriers [37]. Considering the role of monocytes in the bone regeneration process, we used THP1-Blue™ human monocytes (InvivoGen, San Diego, CA, USA) as a sensitive bioindicator of the ability of composites to induce proinflammatory activation of these cells. THP1-Blue™ cells, like other monocytes, respond to ligands, such as peptides, endotoxins, and glycoconjugates, via toll-like receptors (TLR-2, TLR-4, TLR-5, TLR-6, and TLR-8). Upon TLR stimulation, NF-κB is activated; subsequently, the reporter enzyme is secreted into the cell culture milieu. In contrast to studies based on peripheral blood monocytes, this protocol does not require ethical commission approval, does not depend on genetic variability of donors, and is repeatable and quantitative.

**Figure 8:**
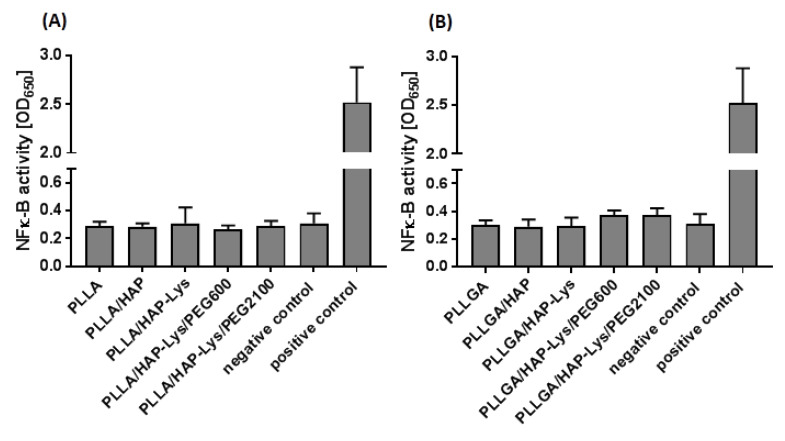
The activation of NF-κB transcription factor in THP1-Blue™ in monocytes incubated for 24 h with (**A**) PLLA- and (**B**) PLLGA-based composites in comparison with monocytes stimulated with lipopolysaccharide (LPS) of *Escherichia coli* (positive control) or cell culture medium (negative control). The secreted embryonic alkaline phosphatase release following toll-like receptor stimulation and NF-κB induction was quantified spectrophotometrically (OD = 650 nm) after enzymatic substrate conversion. Data are presented as mean ± SD of three separate experiments (six replicates of each assay).

Results obtained in the immunocompatibility in vitro assay using THP1-Blue^TM^ cells showed that PLLA- and PLLGA-based composites did not stimulate human monocytes (Figure 8). The obtained values of secreted embryonic alkaline phosphatase (SEAP) concentrations remained on the level of unstimulated cultures (0.308 ± 0.07). Thus, SEAP production by monocytes in response to PLLA, PLLA/HAP, PLLA/HAP-Lys, and PLLA/HAP-Lys/PE600 was 0.286 ± 0.03, 0.284 ± 0.02, 0.308 ± 0.115, 0.266 ± 0.02, and 0.287 ± 0.03, respectively. Similarly, the PLLGA composites (PLLGA, PLLGA/HAP, PLLGA/HAP-Lys, PLLGA/HAP-Lys/PEG600, and PLLGA/HAP-Lys/PEG2000) did not activate monocytes to induce NF-kB transcription factor (0.299 ± 0.03, 0.289 ± 0.05, 0.295 ± 0.05, 0.372 ± 0.03, and 0.375 ± 0.04, respectively). In comparison, strong activation of THP1-Blue™ NF-κB monocytes was observed in cultures stimulated with lipopolysaccharide (LPS) *Escherichia coli* (100 ng/mL), which is a well-established bacterial stimulus (2.52 ± 0.360).

Since biologically active compounds such as peptides might lead to the pathological activation of monocytes, our findings on the lack of immunostimulatory properties of PLLA and PLGA composites containing hydroxyapatite with l-lysine supported their biosafety, not only with regard to lack of cytotoxicity, but also in relation to innate immunity. Conversely, recent studies on biomaterials for bone regeneration suggested mild activation of monocytes in the milieu of implantation, especially in the context of the p58 mitogen-activated protein kinase (MAPK) pathway supporting angiogenesis and bone regeneration [38,39]. These opposing findings on the role of monocyte activation in bone regeneration might be explained by monocyte polarization into proinflammatory (M1) or proregenerative (M2) phenotype. The monocyte polarization process depends on the type of bone fraction, type of biocomposite, host immune defense mechanisms, cytokine microenvironment, as well as the presence of bacteria and their antigens [37,40,41].

More importantly, the lack of composite-mediated activation of monocytes provides information on the amount of endotoxin contaminants, which, according to the Food and Drug Agency Guidance as well as the European Medicines Agency, cannot be present in biomaterials that have contact with human blood/tissues, in amounts higher than 0.25 EU. Based on our optimization study, the activation of THP1-Blue™ monocytes with 0.25 EU of endotoxin resulted in a significant increase in absorbance to 0.600 (OD = 650 nm, optical density), which was not observed for any of the tested composites.

## 3. Materials and Methods

Poly(l-lactide) Resomer L 207 S (PLLA) and poly(l-lactide-co-glycolide) Resomer LG 855 S (PLLGA) supplied by Evonik (Evonik Industry AG, Essen, Germany), and synthetic hydroxyapatite powder (HAP) with a particle size of <200 nm from Sigma-Aldrich (Darmstadt, Germany) were used in our research. Two carboxyl-terminated PEGs with different molecular weights and chemical formulas (Table 4) were obtained from Sigma-Aldrich (Darmstadt, Germany). Polyethylene glycol monomethyl ether succinate with a relative molecular mass of around 2100 g/mol (PEG2100) and *O*-(2-carboxyethyl)-*O′*-methyl-undecaethylene glycol (PEG600) were used. The chemical structures and properties of PEG2100 and PEG600 are presented in Table 4. Crystallized l-lysine, 1,1′-carbonyldiimidazole (CDI), and dry dichloromethane (Sigma-Aldrich, Darmstadt, Germany) were used as received. Hydroxyapatite was dried at 110 °C for 24 h in a vacuum prior to use.

### 3.1. Modification of Hydroxyapatite with l-Lysine

A dispersion of 0.5 g of hydroxyapatite in 5 mL of distilled water was sonicated for 10 min. We added 0.146 g of l-lysine to the suspension, which was magnetically stirred for 24 h at room temperature (RT). The powder was separated by centrifuging and was dried at 110 °C under vacuum for 24 h.

### 3.2. PEG Grafting to HAP Modified with l-Lysine

PEGs were grafted to l-lysine-modified HAP particles with CDI at a molar ratio of the first order amine group of HAP-Lys:PEG:CDI (1:1:1.3). Polyethylene glycols with a carboxyl end group were activated with CDI coupling agent in 10 mL of dichloromethane (DCM) for 2 h at room temperature under argon atmosphere. A dispersion of hydroxyapatite modified with l-lysine (0.05 g) in 10 mL of DCM was sonicated and added to the PEG solution. The suspension was mixed at room temperature for 4 h under argon. The product was separated by centrifuging and drying at 110 °C for 24 h.

### 3.3. Preparation of Composites

A series of PLLA- and PLLGA-based composites were prepared with 10 wt.% unmodified HAP, l-lysine-modified HAP, and HAP grafted with PEG600 and PEG2100 with the assistance of l-lysine. The general procedure of preparing composites with PEG-modified HAP particles followed the stages of: activation of PEG with CDI, grafting of PEG to l-lysine-modified HAP particles, and addition of PLLA or PLLGA solution. These stages were performed in “one pot” in DCM under an argon atmosphere. Composite films were formed by casting final dispersions and drying. The detailed procedure of composite film preparation is described below for PLLA/HAP-Lys/PEG2100 as an example.

### 3.4. PLLA/HAP-Lys/PEG2100 Composite Film Preparation

We dissolved 0.034 g of PEG2100 in 5 mL of DCM. After the addition of 3.58 mg of CDI, the solution was stirred for 2 h at room temperature under an argon atmosphere. We added 0.05 g of HAP particles modified with l-lysine and the dispersion was sonicated for 5 min under argon and stirred for 4 h. A solution of 0.756 g of PLLA in 10 mL of DCM was added. The final dispersion was sonicated for 5 min and stirring was continued for 10 min. The dispersion was cast on a Petri dish (90 mm in diameter) and was left for 24 h at RT for evaporation of DCM. The composite film was additionally dried for 4 h at 40 °C in a vacuum drier. The average thickness of the composite films after drying was 35 µm.

### 3.5. Fourier-Transform Infrared Spectroscopy

Hydroxyapatite particles surface-modified with l-lysine and PEG-grafted hydroxyapatite particles were analyzed by means of FTIR spectroscopy in the ATR mode. ATR spectra were recorded on a Nicolet iZ10 spectrometer (Thermo Scientific, Waltham, MA, USA). Spectra were recorded at the wave number range of 4000–550 cm^−1^ with a spectral resolution of 4 cm^−1^, with 32 co-added scans.

### 3.6. Thermogravimetry

A thermogravimetric analysis was performed for unmodified and surface-modified HAP particles. Thermogravimetric measurements were recorded using the TGA/DSC1 Mettler Toledo thermobalance (Greifensee, Switzerland). Samples were heated at a rate of 10 °C/min from 25 to 800 °C under 60 mL/min air flow. For the purpose of data presentation, the TGA curves were exported to OriginPro 64 (v.9.0) (OriginLab Corportaion, Northampton, MA, USA) as ASCII files.

### 3.7. Optical Microscopy

Optical images of composite films were taken with the Keyence VHX-5000 digital microscope (Keyence, Osaka, Japan).

### 3.8. Scanning Microscopy

Scanning electron microscopy (SEM) coupled with energy-dispersive X-ray diffraction (EDS) (Phenom ProX; Thermo Scientific, Waltham, MA, USA) was used to observe the morphology and elemental composition of hydroxyapatite powder samples with and without functionalization. For each sample, 10 independent measurements were recorded. The elemental composition as evaluated by EDS was analyzed on weight and atomic percentage base for all samples. Information on calcium-to-phosphate molar and atomic ratios is provided. In order to determine the aggregability of hydroxyapatite powder samples, a quantitative analysis of the average size of particles was performed based on SEM images in ImageJ software (v.1.8.0, National Institutes of Health, USA). Particle size (surface area) was measured following bandpass filtering and thresholding, whereas processing parameters were selected individually for each image due to differences in scale, quality, and brightness.

### 3.9. Water Contact Angle

Water contact angle measurements were conducted using a Surftens universal goniometer (OEG Gesellschaft für Optik, Elektronik & Gerätetechnik mbHGmbH, Frankfurt (Oder), Germany). At least eight measurements for each sample were performed and data were used to calculate the final average results, as well as standard deviation (Table S1 in Supplementary Materials).

### 3.10. Differential Scanning Calorimetry

Differential scanning calorimetry (DSC) measurements were recorded using a Mettler Toledo DSC1 (Greifensee, Switzerland) system, coupled with a Huber TC100 intracooler (Offenburg, Germany). The instrument was calibrated using indium (T_m_ = 156.6 °C, ΔH_m_ = 28.45 J/g) and zinc (T_m_ = 419.7 °C, ΔH_m_ = 107.00 J/g) standards. Samples (~3.5 mg) were measured in 40 mL aluminum pans under a constant nitrogen purge (60 mL/min) from 0 to 200 °C. After the heating cycle, the samples were thermally equilibrated at 200 °C for 5 min and cooled to 0 °C. A second heating scan was also performed. Both heating and cooling rates were set to 5 °C/min. The glass transition temperature from heating and cooling scans was taken as the inflection point in the heat flux curve. Experimental data were processed using the generic STAR^e^ (Mettler Toledo, Greifensee, Switzerland) computer program. For the purpose of data presentation, the DSC profiles were exported to OriginPro 64 (v.9.0) (OriginLab Corportaion, Northampton, MA, USA) as ASCII files.

### 3.11. In Vitro Cytocompatibility

#### 3.11.1. Cell Culture Conditions

The influence of composites on cell viability was assessed according to ISO 10993-5:2009 (Biological evaluation of medical devices—Part 5: Tests for *in vitro* cytotoxicity) toward two cell lines. The L929 (CCL-1™) mouse skin fibroblasts and hFOB 1.19 (CRL-11372™) human fetal osteoblastic cell line were obtained from the American Type Culture Collection (ATCC, Manassas, VA, USA). Prior to experiments, fibroblasts were cultured in Roswell Park Memorial Institute (RPMI)-1640 medium supplemented with 10% heat-inactivated fetal bovine serum (FBS; HyClone Cytiva, Marlborough, MA, USA), penicillin (100 U/mL), and streptomycin (100 μg/mL) (Sigma-Aldrich, Darmstadt, Germany) in a humidified 5% CO_2_ atmosphere at 37 °C (cell culture incubator). Osteoblasts were cultured in a 1:1 mixture of phenol-free Dulbecco’s modified Eagle’s medium and Ham’s 12-F medium (Gibco, Waltham, MA, USA) supplemented with 10% FBS (HyClone Cytiva, Marlborough, MA, USA) and 0.3 mg/mL G418 (Sigma-Aldrich, Darmstadt, Germany) in a cell culture incubator. For subculturing, confluent cell monolayers were detached from the culture vessel with 0.5% trypsin-EDTA solution (Gibco, Waltham, MA, USA) and suspended in culture medium. The cell viability and density were established using a Bruker chamber (Blaubrand, Wertheim, Germany) and trypan blue exclusion assay, respectively. The cells were used in the experiments only if cell viability exceeded 95%.

#### 3.11.2. Direct Contact Cytotoxicity Assay

The cells adjusted to a density of 4 × 10^5^ cells/mL (for hFOB 1.19) or 2 × 10^5^ cells/mL (for L929) were transferred (100 µL/well) into 96-well culture plates (Nunclon Delta Surface, Nunc, Rochester, NY, USA) and incubated overnight (as previously described) to recreate cell monolayers. After incubation, cell cultures were observed using an inverted microscope (Motic AE2000, Barcelona, Spain) to ensure that the confluent and homogeneous monolayers had formed and to exclude contamination. The medium was replaced with 100 μL of fresh culture medium, and composites, cut into pieces corresponding to one-tenth of the well surface area, were added to selected wells (in six replicates). After overnight incubation, the condition of cell monolayers was evaluated under a light microscope and any changes in their morphology were recorded. The cell cultures in medium without the tested materials were used as a positive control of viability. To quantify the viability of cells, 20 μL of 3-(4,5-dimethylthiazol-2-yl)-2,5-diphenyltetrazolium bromide (Sigma-Aldrich, Darmstadt, Germany) was added to each well and incubation was continued for the next 4 h. In the next step, the plates were centrifuged at 450 g for 10 min, and the supernatants were removed and replaced with 100 μL of DMSO (dimethyl sulfoxide). After few minutes of incubation at room temperature on a rotor vortex, 200 μL of liquid from each well was transferred to a separate 96-well plate and the absorbance was measured at 570 nm using the Multiskan EX reader (Thermo Scientific, Waltham, MA, USA).

### 3.12. Proinflammatory Assay

The THP1-Blue™ NF-κB human monocytes, carrying NF-κB-inducible SEAP reporter construct, obtained from InvivoGen (San Diego, CA, USA), were used to determine the activation of the NF-κB signal transduction pathway via tested materials. This cell line is a sensitive biosensor of innate immunity cell activation. The induction of the NF-κB transcription factor results in embryonic alkaline phosphatase (SEAP) secretion into culture medium. The concentration of the enzyme corresponds with the intensity of stimulation. Human monocytes were cultured in RPMI medium supplemented with 10% heat-inactivated FBS, 25 mM 4-(2-hydroxyethyl)-1-piperazineethanesulfonic acid (HEPES), 100 U/mL penicillin, 100 μg/mL streptomycin, 2 mM glutamine, and selective agents (100 μg/mL normocin and 10 μg/mL blasticidin) at densities below 2 × 10^6^ cells/mL in a humidified 5% CO_2_ atmosphere at 37 °C. THP1-Blue™ NF-κB monocytes were adjusted to 1 × 10^6^ cells/mL and 200 µL of cell suspension was transferred to each well. Then, composites, cut into pieces corresponding to one-tenth of the well surface area, were added to selected wells (in six replicates). The monocytes incubated in medium served as the negative control, whereas monocytes stimulated with LPS *E. coli* (100 ng/mL) were used as a positive control for NF-κB activation. SEAP production was quantified by combining 20 μL of cell-free supernatant with 180 μL QUANTI-Blue™ buffer (InvivoGen, San Diego, CA, USA) and incubated at 37 °C with 5% CO_2_ for 4 h. Absorbance was measured at 650 nm on the Multiskan EX reader (Thermo Scientific, Waltham, MA, USA).

### 3.13. Statistical Analysis

The statistical significance of the differences between groups obtained in biological studies were assessed using the nonparametric Mann–Whitney U test. The differences were considered to be statistically significant when *p* < 0.05. A statistical analysis was performed using the GraphPad Prism 7 software (GraphPad Software, San Diego, CA, USA).

## 4. Conclusions

The main goal of this research was to design novel biocomposites based on PLLA and PLLGA with HAP surface-functionalized HAP particles. HAP was successfully modified with polyethylene glycols of different molecular weights with the assistance of l-lysine, as confirmed qualitatively and quantitatively by FTIR, TGA, and elemental composition analysis. The effect of grafting of PEG molecules, which are miscible with PLLA and PLLGA, on morphology was evidenced by optical microscopy. The modification of HAP with PEG enhanced the dispersion of HAP particles.

The analysis of DSC results indicated the nucleating activity on PLLA melt crystallization exhibited by HAP modified with PEGs, with the efficiency depending on the type of PEGs. We performed a detailed analysis of molecular weight, mass content, and chemical structure of PEG. We found that, in the presence of HAP modified with PEG2100, PLLA crystallized from melt in the α form; for HAP modified with PEG600, a mixture of α’ and α form crystals developed on cooling. This finding is important for the application of composites in regenerative medicine as bone scaffolds. The crystal form of PLLA significantly affects the mechanical properties and exhibits different hydrolytic degradation behavior [42,43,44]. For PLLGA, which has lower crystallization ability than PLLA, nucleation of melt crystallization was achieved only by HAP modified with PEG2100, whereas cold crystallization was enhanced by HAP modified with PEG2100 and PEG600. Significant plasticization of PLLA and PLLGA, confirmed by significant reductions of T_g_ and T_m_, was achieved with a low content of PEGs.

Based on the findings, we concluded that due to hydroxyapatite surface modification, multifunctional HAP particles were created that can play the roles of nucleating agents, plasticizers, and bioactive moieties. By choosing the molecular weight of PEGs, crystallinity degree of PLLA and PLLGA, and polymorphic form of PLLA, the most important factors affecting mechanical properties and degradation behavior can be controlled.

The investigated PLLA- and PLLGA-based composites were found to be non-toxic against the mouse fibroblasts L929 in an MTT reduction assay, and did not decrease the viability of human osteoblasts hFOB in a similar assay. Importantly, all tested materials were confirmed to be non-pyrogenic as they did not cause significant activation of the human THP1-Blue^TM^ cells, a monocyte/macrophage-mediated inflammation sensing system. The l-lysine- and PEG-functionalized biomaterials tested in the current study supported the viability of human osteoblastic progenitor cells. In terms of biosafety and cytocompatibility, the addition of l-lysine to HAP-based bone substitute materials used for bone regeneration was highly beneficial for improving their characteristics. Based on previous reports, l-lysine promotes cell adhesion, similar to poly-l-lysine. Overall, all tested composites demonstrated good cytocompatibility profiles and are promising materials for future development of medical composites that could be used in bone regeneration. The findings provide the initial characteristics of promising materials that will be further explored in advanced in vitro and in vivo experimental setups. In this regard, an analysis of cell adhesion and differentiation, and studies on vascularization of the materials are planned for an upcoming, separate study.

## Figures and Tables

**Table 1:** Elemental Composition and Average Particle Size (Presented as Average and Standard Deviation) of Pure and Modified Hydroxyapatite (HAP) Samples.

	HAP		HAP-Lys		HAP-Lys/PEG600		HAP-Lys/PEG2100	
Element	at%	wt.%	at%	wt.%	at%	wt.%	at%	wt.%
C	39.89 ± 6.25	26.40 ± 5.52	45.15 ± 7.23	32.04 ± 4.92	28.59 ± 7.42	18.47 ± 4.16	34.63 ± 9.79	23.09 ± 8.15
O	44.32 ± 7.30	40.88 ± 7.05	33.10 ± 6.55	31.38 ± 6.21	44.24 ± 9.36	38.21 ± 7.63	38.31 ± 9.24	33.76 ± 10.80
N	-	-	6.49 ± 2.44	5.34 ± 0.51	8.47 ± 0.65	6.38 ± 0.43	8.56 ± 1.01	6.56 ± 0.73
Ca	9.84 ± 1.63	22.23 ± 3.22	8.99 ± 1.87	21.25 ± 4.20	12.03 ± 2.51	25.01 ± 5.19	12.18 ± 4.66	26.08 ± 7.96
P	5.86 ± 0.88	10.32 ± 1.32	4.93 ± 0.93	8.78 ± 1.57	6.51 ± 0.99	10.82 ± 1.42	6.41 ± 1.89	10.56 ± 2.38
Ca/P molar ratio	1.67		1.87		1.79		1.91	
Ca/P atomic ratio	1.68		1.89		1.85		1.90	
Particle average size (µm^2^)	11.36 ± 2.90		8.89 ± 2.12		5.48 ± 1.21		8.64 ± 3.92	

**Table 2:** Thermal Parameters from the Cooling DSC Curves of PLLA and PLLGA Sample Series.

Sample	T_c_^onset^ (°C)	T_c_ (˚C)	ΔH_c_ (J/g)	T_g_ (°C)
PLLA	114.0	102.5	11.9	59.7
PLLA/HAP	112.8	102.2	23.8	58.6
PLLA/HAP-Lys	112.0	102.2	24.0	61.5
PLLA/HAP-Lys/PEG600	111.2	104.0	32.1	43.7
PLLA/HAP-Lys/PEG2100	116.5	108.2	37.9	35.2
PLLGA	-	-	-	56.3
PLLGA/HAP	-	-	-	56.2
PLLGA/HAP-Lys	-	-	-	55.5
PLLGA/HAP-Lys/PEG600	-	-	-	47.0
PLLGA/HAP-Lys/PEG2100	118.3	100.9	10.8	28.7

**Table 3:** Thermal Parameters from Second Heating DSC Curves of PLLA and PLLGA Sample Series.

Sample	T_g_ (°C)	T_cc_^onset^ (°C)	T_cc_ (°C)	ΔH_cc_ (J/g)	T_α’-α_ (°C)	ΔH_α’-α_ (J/g)	T_m_ (°C)	ΔH_m_ (J/g)
PLLA	62.6	97.2	108.9	25.7	160.9	6.4	177.9	46.4
PLLA/HAP	61.4	84.9	99.1	8.7		6.1	170.8	42.7
PLLA/HAP-Lys	63.1	84.3	97.8	6.7	159.5	5.7	170.6	41.8
PLLA/HAP-Lys/PEG600	57.0	76.5	115.8	2.0	161.5	4.0	170.9	43.3
PLLA/HAP-Lys/PEG2100	46.0	-	-	-	-	-	172.0	41.2
PLLGA	59.5	-	-	-	-	-	-	-
PLLGA/HAP	59.6	-	-	-	-	-	-	-
PLLGA/HAP-Lys	59.62	-	-	-	-	-	-	-
PLLGA/HAP-Lys/PEG600	50.1	98.39	109.0	28.1	-	-	161.7	24.6
PLLGA/HAP-Lys/PEG2100	41.6	86.68	97.5	14.8	-	-	161.8	28.7

**Table 4:** Properties of Polyethylene Glycols Used in This Study.

Material	Chemical Formula	T_g_ [°C]	T_m_ [°C]	Molecular Weight *
PEG600	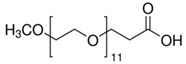	−64.4	20.2	588.86 g/mol
PEG2100	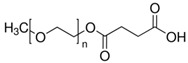	−33.1	53.2	~2100 g/mol

* As specified by the supplier (Sigma-Aldrich, Darmstadt, Germany)

## Supplementary Materials

The following are available online at https://www.mdpi.com/1422-0067/21/18/6711/s1, Figure S1: ATR-FTIR spectra of HAP (blue), L-lysine (black) and HAP modified with L-lysine (red), Figure S2: TGA curves of PEG 2100 and L-lysine, Table S1: Values of water contact angle measurements.

## Abbreviations

LD	linear dichroism
ATR	attenuated total reflectance
CDI	1,1′-carbonyldiimidazole
DCM	dichloromethane
DMSO	dimethyl sulfoxide
DSC	differential scanning calorimetry
EDS	energy-dispersive X-ray diffraction
EDTA	ethylenediaminetetraacetic acid tetrasodium salt dihydrate
FBS	fetal bovine serum
HAP	hydroxyapatite
HEPES	4-(2-hydroxyethyl)-1-piperazineethanesulfonic acid
LPS	lipopolysaccharide
Lys	l-lysine
MAPK	mitogen-activated protein kinase
MTT	3-(4,5-dimethylthiazol-2-yl)-2,5-diphenyltetrazolium bromide
NF-κB	nuclear factor kappa-light-chain-enhancer of activated B cells
OD	optical density
PEG2100	*O*-methyl-*O′*-succinylpolyethylene glycol
PEG600	*O*-(2-carboxyethyl)-*O′*-methyl-undecaethylene glycol
PLLA	poly(l-lactide)
PLLGA	poly(l-lactide-co-glycolide)
RANKL	Receptor Activator for Nuclear Factor-κB Ligand
RT	room temperature
SEAP	secreted embryonic alkaline phosphatase
SEM	scanning electron microscopy
TGA	thermogravimetry
TLR	toll-like receptor
T_c_	peak temperature of melt crystallization exotherm
T_c_^onset^	temperature of onset of melt crystallization
T_cc_	peak temperature of cold crystallization exotherm
T_cc_^onset^	temperature of onset of cold crystallization
T_g_	glass transition temperature
T_m_	peak temperature of melting endotherm
T_α’-α_	temperature of α’-α phase transition
ΔH_c_	enthalpy of melt crystallization
ΔH_cc_	enthalpy of cold crystallization
ΔH_α’-α_	enthalpy of α’-α phase transition
ΔH_m_	enthalpy of melting
