# Supplementary material for: Influence of Hydroxyapatite Surface Functionalization on Thermal and Biological Properties of Poly(l-Lactide)- and Poly(l-Lactide-co-Glycolide)-Based Composites
Source: Int J Mol Sci. 2020 Sep 13;21(18):6711. doi: 10.3390/ijms21186711 (PMC7556045; doi:10.3390/ijms21186711)
Supplement: Supplementary file 1 [file ijms-21-06711-s001.pdf]

International Journal of *Molecular Sciences*

MDPI

Supplementary Materials

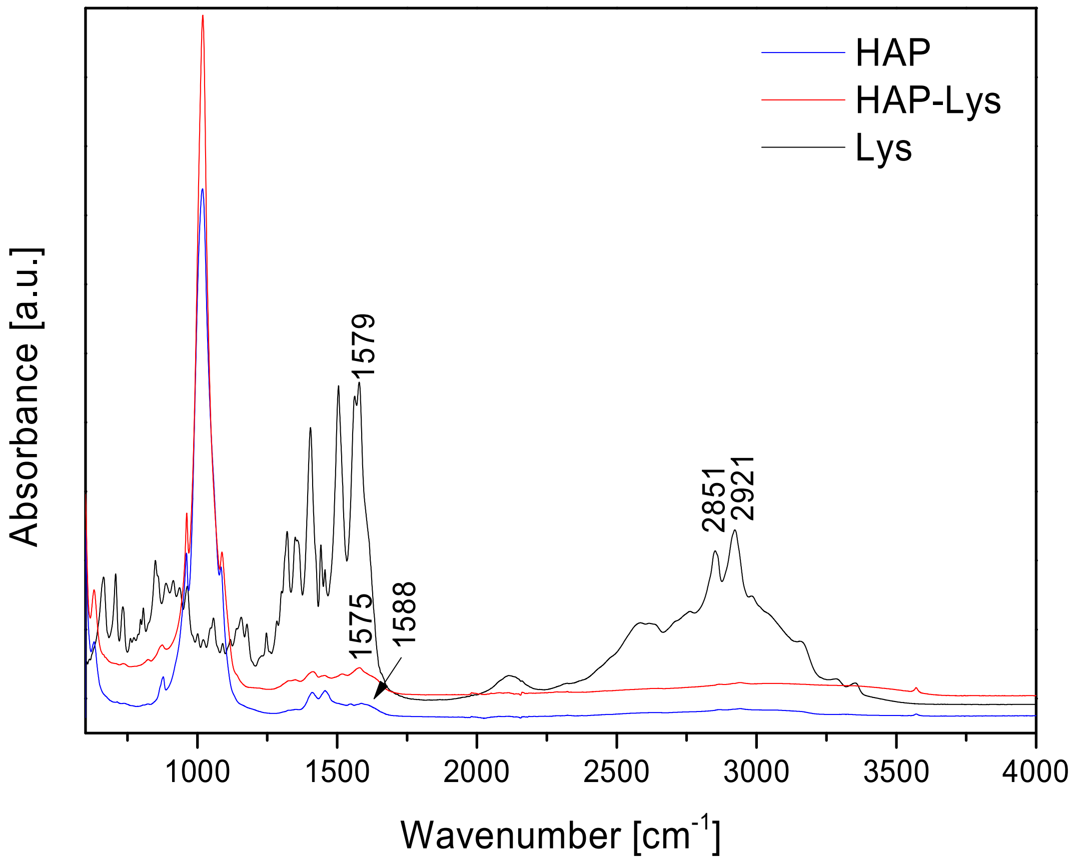

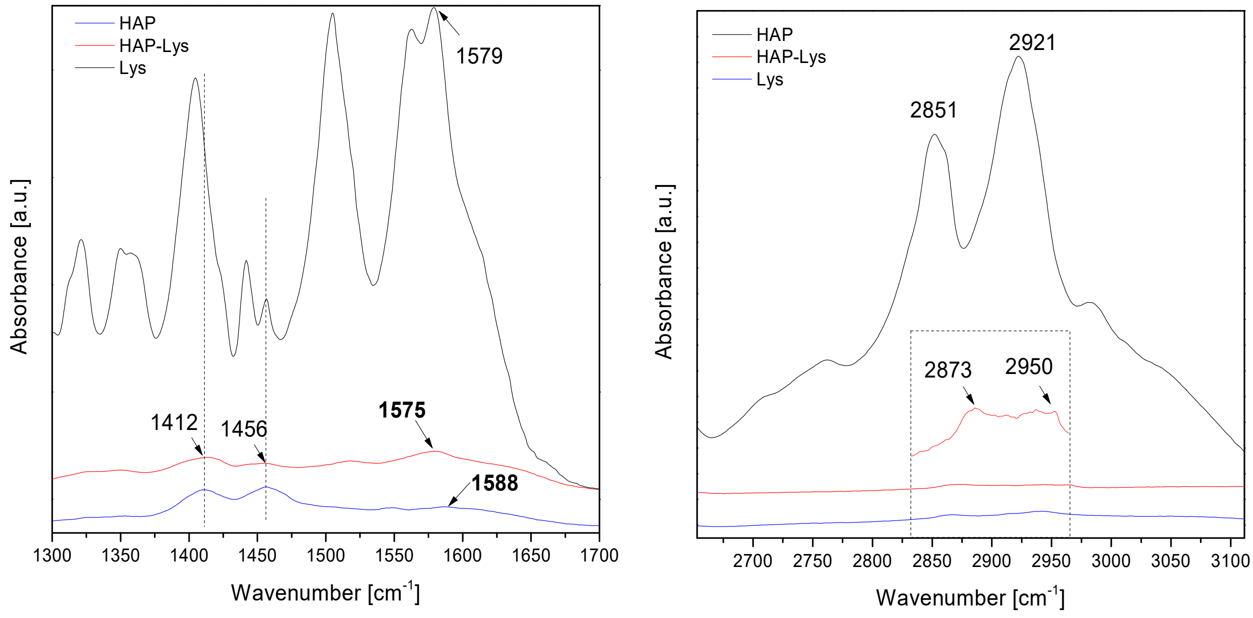

Figure S1. ATR-FTIR spectra of HAP (blue), L-lysine (black) and HAP modified with L-lysine (red).

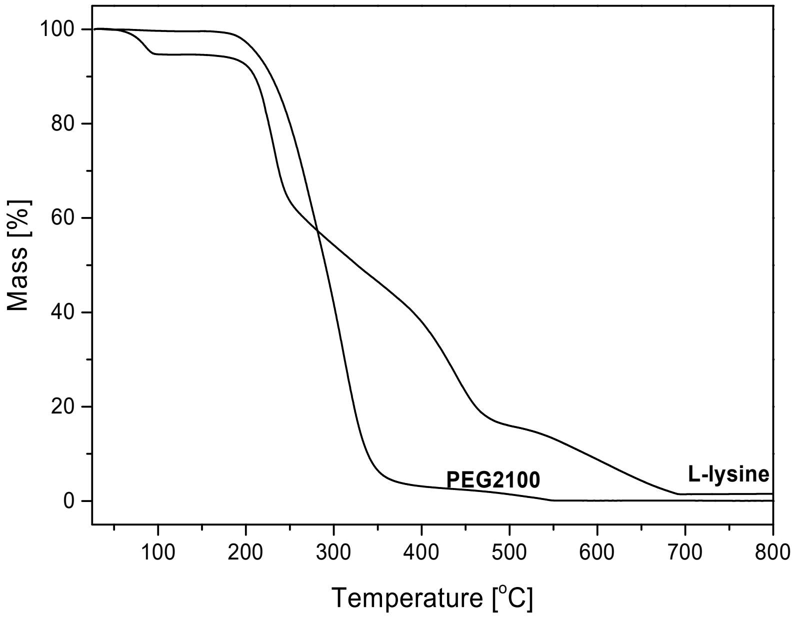

Figure S2. TGA curves of PEG 2100 and L-lysine.

Table S1. Values of water contact angle measurements.

**Sample**	**PLLA**	**PLLA/HAP**	**PLLA/HAP-Lys**	**PLLA/HAP-Lys/PEG600**	**PLLA/HAP-Lys/PEG2100**
Water contact angle [°]	66.2	66.4	60.1	60.1	52.3
	66.2	63.8	57.7	52.2	46.3
	66.7	64.2	59.0	47.8	43.5
	61.3	61.0	59.1	52.3	48.5
	65.3	62.6	62.2	60.7	49.9
	69.1	67.4	59.9	49.1	52.0
	69.5	66.0	59.1	50.8	46.5
	59.7	67.2	62.6	48.2	53.5
Average value	**65.5**	**64.8**	**60.0**	**52.7**	**49.1**
Standard deviation	3.2	2.2	1.6	4.7	3.3
**Sample**	**PLLGA**	**PLLGA/HAP**	**PLLGA/HAP-Lys**	**PLLGA/HAP-Lys/PEG600**	**PLLGA/HAP-Lys/PEG2100**
Water contact angle [°]	62.1	59.1	53.0	54.6	50.2
	64.8	62.7	57.8	56.0	45.2
	62.8	57.7	60.5	52.2	42.6
	63.5	57.3	55.4	57.9	56.1
	63.6	70.1	58.7	57.6	45.3
	59.4	63.5	58.3	57.8	50.8
	61.6	60.5	56.7	56.8	51.6
	63.5	57.3	59.7	51.7	43.0

Average value	**62.7**	**61.0**	**57.5**	**55.6**	**48.1**
Standard deviation	1.5	4.1	2.3	2.3	4.5
